# Role of Fisetin in the Mammalian Reproductive System

**DOI:** 10.3390/vetsci13050438

**Published:** 2026-04-30

**Authors:** Yilong Chen, Xiaogang Huang, Zhihong Zhao, Ronggen Wang, Ruiyan Liu, Jinhao Chen, Li Li, Minhua Hu, Hengxi Wei, Shouquan Zhang

**Affiliations:** 1 State Key Laboratory of Swine and Poultry Breeding Industry, Guangzhou 510642, China; 2 National Engineering Research Center for Breeding Swine Industry, Guangzhou 510642, China; 3 Provincial Key Lab of Agroanimal Genomics and Molecular Breeding, College of Animal Science, South China Agricultural University, Guangzhou 510642, China; 4 Guangzhou General Pharmaceutical Research Institute Co., Ltd., National Canine Laboratory Animal Resources Center, Guangzhou 510240, China; 5 Guangzhou Baiyunshan Beagle Biotechnology Co., Ltd., Guangzhou 510240, China

**Keywords:** Fisetin, Polyphenols, gamete quality, oxidative stress, livestock fertility

## Abstract

Fisetin, a flavonoid widely present in natural plants, exhibits diverse pharmacological activities including antioxidant, anti-inflammatory, anti-apoptotic, and anti-aging effects. Its protective roles within the mammalian reproductive system have gained increasing attention in reproductive medicine and natural product research. This review provides a narrative overview of the regulatory and protective effects of Fisetin on both female and male reproductive systems, with a focus on gonadal function, the reproductive microenvironment, and endocrine regulation. Key molecular mechanisms, such as antioxidant, anti-inflammatory, and metabolic pathways, are discussed. Additionally, challenges related to low bioavailability and the translational potential in both clinical and agricultural settings are considered. By consolidating current knowledge, this review aims to provide a foundation for future research and the development of natural interventions for reproductive health.

## Simple Summary

Mammalian reproductive health faces growing threats from pollution, lifestyle, and aging, leading to conditions like infertility. Current medical treatments can be complex and have side effects. Fisetin is a natural compound found in many fruits and vegetables, such as strawberries and apples. This review examines the potential of Fisetin to protect and improve reproductive health in both females and males. We summarize scientific studies showing that Fisetin can help maintain healthy ovaries and testes, improve the quality of eggs and sperm, and balance reproductive hormones. These beneficial effects are mainly due to Fisetin’s ability to act as a strong antioxidant and anti-inflammatory agent inside cells. While results from animal studies are promising, a major challenge is that Fisetin is poorly absorbed by the body. Future research is needed to develop better ways to deliver Fisetin and to confirm its benefits in humans and livestock. This natural compound holds promise as a safe, supplementary approach for supporting fertility and reproductive well-being.

## 1. Introduction

### 1.1. The Importance of Mammalian Reproductive System Health and Its Challenges

The mammalian reproductive system plays a crucial role in ensuring species continuation, and reproductive health directly determines population survival and individual fertility. In recent years, environmental pollution, dietary shifts, and lifestyle changes have placed unprecedented stress on mammalian (including human) reproductive systems, contributing to a rise in disorders such as menstrual irregularities, premature ovarian insufficiency (POI), and infertility [1]. Studies indicated that the prevalence of reproductive endocrine disorders like Polycystic Ovary Syndrome (PCOS), endometriosis, testicular injury, and Oligospermia is rising annually [2,3], and the incidence of infertility also shows a year-by-year upward trend according to a 2025 global burden of disease study [4].

Despite advances in reproductive treatments, including in vitro fertilization (IVF) and hormone replacement therapy, these approaches have inherent limitations. For instance, IVF success rates are relatively low, and the treatment process is complex and expensive; hormone therapy, on the other hand, may be associated with various side effects under specific clinical contexts, such as an increased risk of breast cancer, uterine cancer, and venous thromboembolism in certain populations [5]. Consequently, finding a natural, safe, and side-effect-free therapeutic approach has become a significant focus in the field of reproductive medicine.

### 1.2. Chemical Properties, Sources, and Basic Pharmacological Activities of Fisetin

Fisetin is a natural flavonoid compound widely found in plants such as strawberries, apples, mangoes, cucumbers, and onions [6]. Its chemical name is 3,3′,4′,7-tetrahydroxyflavone, with the molecular formula C15H10O6 and a molecular weight of 286.24. Its chemical structure was first identified by the scientist Schmidt in 1833 from an extract of Venetian sumac [7], and subsequent studies have confirmed its widespread presence in various natural plants. It exhibits potent biological activities, including antioxidant [8] and anti-inflammatory effects [9]. It has been reported to scavenge reactive oxygen species (ROS) and inhibit inflammatory responses in the body. It reduces oxidative stress and inflammation, thereby protecting cells from damage.

Furthermore, studies indicate that Fisetin is also involved in biological processes such as anti-tumor [10], anti-aging [11], and anti-apoptotic effects [12] in various in vitro and animal models, and it has been the subject of growing research interest for the prevention and treatment of various diseases. Consequently, the potential protective effects of Fisetin on the nervous system, immune system, and reproductive system have attracted increasing scientific attention [13].

### 1.3. Origins and Current Status of Fisetin Research in Reproductive Biology

With the in-depth exploration of Fisetin’s pharmacological activities, studies into its protective effects and related mechanisms within the mammalian reproductive system have gradually emerged. This has become a convergent hotspot in the fields of reproductive medicine and natural product studies, offering new perspectives for addressing the challenges facing mammalian reproductive health. Existing studies have preliminarily confirmed that Fisetin, leveraging its core pharmacological activities such as antioxidant, anti-inflammatory, and anti-apoptotic effects, may protect both the mammalian reproductive systems through multiple targets and pathways. Furthermore, due to its safety as a natural product, it offers unique advantages compared to traditional interventions like hormone therapy.

In females, the protective effects of Fisetin have been primarily investigated in the context of ovarian function, oocyte quality, and reproduction-related diseases. For instance, a study using mouse models found that Fisetin can activate the sirtuin 1 (Sirt1) signaling pathway and enhance antioxidant capacity, thereby delaying post-ovulatory oocyte aging [14]. In laying hen models, Fisetin has been observed to promote ovarian cell proliferation and improve ovarian redox state [15]. Furthermore, in common reproductive endocrine disorders like Polycystic Ovary Syndrome (PCOS), Fisetin may modulate glucolipid metabolism disorders, ameliorate insulin resistance, balance reproductive hormone levels, inhibit the activation of inflammatory signaling pathways, and alleviate ovarian pathological damage.

In males, studies on Fisetin have primarily focused on testicular function and sperm quality. In rat models of oligoasthenospermia, Fisetin was shown to reduce spermatogenic cell apoptosis and improve sperm count and motility [16]. In a rat model of arsenic-induced reproductive toxicity, Fisetin mitigated oxidative stress-induced damage to testicular tissue [17].

Despite this progress, several study gaps remain. Current studies are largely concentrated on rodent models, with a notable lack of clinical studies in the human reproductive system. Most studies report positive effects without comparing negative or null findings, and few address the reproducibility or model-specific limitations. The molecular mechanisms by which Fisetin regulates reproductive system functions are not yet fully elucidated, and its efficacy across different reproductive disorders remains to be defined.

It is worth noting that although the current study on Fisetin in the reproductive field has primarily focused on human disease models, the underlying molecular mechanisms are highly conserved across mammals. This provides a theoretical foundation for exploring whether these studies’ findings could be extended to the reproductive practices of economically important animals such as pigs, cattle, and sheep, though such applications would require direct experimental validation [18,19,20,21,22]. Accordingly, a distinguishing feature of this review is its integrated analysis of translational opportunities across both clinical and agricultural contexts, an aspect rarely addressed in existing Fisetin reviews.

Given the current state of studies, reviewing the roles and mechanisms of Fisetin in the mammalian reproductive system is of great significance for advancing the field. This review aims to integrate recent studies’ progress, elucidate the protective effects and molecular mechanisms of Fisetin within a unified conceptual framework, and discuss its potential applications and the challenges associated with its clinical and translational development.

## 2. Protective and Regulatory Effects of Fisetin on the Mammalian Reproductive System

### 2.1. Effects on the Female Reproductive System

#### 2.1.1. Ovarian Function and Follicular Development

Regarding ovarian function and follicular development, the core role of Fisetin lies in mitigating oxidative stress and cellular senescence. Oxidative stress is known to impair follicular development and hormonal balance and is associated with premature ovarian insufficiency (POI) and age-related decline in ovarian reserve [23]. Chemically induced ovarian dysfunction models, such as those involving chemotherapy, further exacerbate oxidative damage, leading to a compromised ovarian microenvironment and reduced fertility potential [24]. In models of natural aging or chemically induced ovarian decline, Fisetin treatment has been reported to effectively reduce ROS accumulation and lipid peroxidation levels within the ovary, thereby improving ovarian reserve function. Studies have found that Fisetin upregulates Sirt1 in aged oocytes, increasing the expression levels of the mitochondrial transcription factor A (Tfam) as well as the mitochondrial genes Co2 and Atp8, ultimately slowing oocyte aging [14]. Furthermore, Fisetin has been identified as a potent inhibitor of the senescence-associated secretory phenotype (SASP) and a senolytic agent [11]. In the ovary, it may selectively eliminate senescent granulosa cells accumulated due to aging or stress. This action mitigates the adverse effects of the senescent microenvironment on follicular development, providing a novel strategy for improving oocyte quality in women of advanced age or with diminished ovarian reserve. In PCOS models, Fisetin has been reported to improve insulin resistance via metabolic signaling pathways and reduce serum androgen levels, thereby promoting normal follicular development and ovulation [25]. In an ovarian ischemia–reperfusion injury model, Fisetin, by targeting the toll-like receptor-4/myeloid differentiation primary response 88/TNF-receptor associated factor 6 (TLR4/MyD88/TRAF6)signaling pathway, dose-dependently ameliorated rat ovarian weight and hematological parameters, downregulated pro-inflammatory cytokine levels such as tumor necrosis factor-α (TNF-α), interleukin-1β (IL-1β), and interleukin-6 (IL-6), inhibited nuclear factor kappa-B (NF-κB) activation and caspase-3-mediated apoptotic pathways, while simultaneously enhancing the activities of antioxidant enzymes like superoxide dismutase and glutathione, and reducing malondialdehyde accumulation. This resulted in a significant protective effect, alleviating ovarian tissue hemorrhage, edema, and inflammatory infiltration [26]. The ovarian protective mechanisms of Fisetin revealed above have important implications for the reproductive management of high-yielding female livestock (such as dairy cows and sows). Under high-yielding conditions, these animals often experience follicular development arrest, ovulation disorders, and early embryonic loss due to metabolic stress [27,28]. By acting as a multi-pathway modulator, Fisetin could potentially be used as a feed additive or a follicular fluid supplement to improve the ovarian microenvironment and extend reproductive lifespan [29]. However, a comparison across studies reveals inconsistencies. While some models show robust Sirt1 upregulation following Fisetin treatment [14], others report only modest changes in antioxidant enzyme activity without corresponding improvements in follicular survival [26]. These discrepancies may arise from differences in dosage (ranging from 10 to 50 mg/kg), treatment duration, or the severity of oxidative stress induction. Furthermore, most studies rely on acute or subacute injury models, which may not accurately reflect the chronic, low-grade oxidative stress characteristic of age-related ovarian decline. Without long-term studies in healthy aging animals, the translational relevance of these findings remains uncertain. Future studies in animals such as cattle and pigs should validate its practical effects on follicular development, ovulation rate, and embryo production efficiency.

#### 2.1.2. Uterus and Embryo Implantation

Impaired endometrial receptivity is a key factor in embryo implantation failure. Fisetin primarily exerts its effects by improving endometrial receptivity and reducing endometrial pathologies. Studies indicate that flavonoids, including Fisetin, have been shown to inhibit the activation of inflammatory mediators such as NF-κB and activator protein-1 (AP-1), as well as the production of cytokines like IL-1β, TNF-α, and IL-6 [30]. This action highlights their potential in alleviating chronic inflammation and oxidative stress, which are central to the pathogenesis of various diseases. In other words, through this mechanism, Fisetin may mitigate chronic endometrial inflammation, creating a favorable immune environment for embryo implantation.

Current studies emphasize the importance of regulating angiogenesis and apoptosis in the pathogenesis and potential treatment of endometriosis. Numerous studies focus on the modulation of vascular endothelial growth factor (VEGF), a key pro-angiogenic factor involved in the growth of ectopic endometrium. Ricci et al. [31] confirmed that inhibiting VEGF activity using bevacizumab effectively reduces endometrial implant growth in mouse models, indicating the critical role of VEGF in lesion development. Similarly, Cao et al. [32] reported that ginsenoside Rg3 inhibits angiogenesis via the VEGFR-2-mediated PI3K/Akt/mTOR pathway, leading to reduced ectopic endometrial tissue and increased apoptosis, highlighting the therapeutic potential of targeting VEGF signaling pathways. Beyond direct VEGF inhibition, other molecular mechanisms affecting angiogenesis and cell survival have been explored. Zhang et al. [33] investigated the role of microRNA-138, which regulates VEGF expression through the NF-κB signaling pathway, thereby influencing inflammation and apoptosis in endometriosis. These findings suggest that miRNA-mediated VEGF regulation may be a crucial factor in disease progression.

Furthermore, the induction of apoptosis in ectopic endometrial cells has been a research focus. Mazumdar et al. [34] studied the impact of oxidative stress and apoptotic markers, demonstrating that factors inducing oxidative stress may reduce lesion proliferation. Accumulating evidence also points to the role of miRNAs in modulating key signaling pathways. Okamoto et al. [35] identified miR-210 as a promoter in endometriosis pathogenesis by activating the STAT3 pathway, which is associated with cell proliferation and survival. Liu et al. [36] further elucidated that miR-199a-5p inhibits epithelial–mesenchymal transition (EMT) by targeting Zinc finger E-box binding homeobox 1 (ZEB1) via the Phosphatidylinositol 3-kinase/Protein kinase B/Mechanistic target of rapamycin (PI3K/Akt/mTOR) pathway, a pathway also involved in angiogenesis and apoptosis regulation.

It is important to note that existing studies have not directly tested Fisetin in models of endometriosis. However, given its structural similarity to other bioactive flavonoids and its established anti-inflammatory properties, it is plausible to hypothesize that Fisetin may exert beneficial effects. The collective evidence from related compounds indicates that Fisetin possesses the potential to inhibit VEGF expression and induce apoptosis, either through direct action or via microRNA regulation [10,37,38]. We therefore propose that these represent candidate mechanisms that could contribute to inhibiting the growth of ectopic endometrial tissue, aligning with therapeutic strategies for endometriosis.

The health of the uterine microenvironment directly determines the success rate of embryo implantation, which in livestock production translates into improved conception rates and litter sizes [39]. The anti-inflammatory and pro-angiogenic regulatory effects of Fisetin suggest its potential to improve endometrial receptivity in domestic animals, particularly in female livestock experiencing repeated insemination failure or a high incidence of endometritis. Fisetin has been shown to alleviate lipopolysaccharide-induced endometritis in cattle by inhibiting the NF-κB pathway, significantly reducing the expression of inflammatory cytokines and improving the endometrial microenvironment [40]. Combined with emerging mechanisms such as miRNA regulation, future studies could explore the application of Fisetin in recipient animals for embryo transfer, aiming to enhance embryo implantation rates and pregnancy maintenance.

#### 2.1.3. Reproductive Endocrine Axis

The regulatory effect of Fisetin on the hypothalamic–pituitary–ovarian (HPO) axis reflects its systemic impact on the reproductive system. In preclinical models of endocrine disorders such as PCOS, Fisetin not only acts directly on the ovaries to improve follicular development but also may indirectly regulate HPO axis function by influencing central metabolic sensing pathways. Research has found that Fisetin has been reported to activate AMP-activated protein kinase (AMPK) and its upstream kinase LKB1, thereby upregulating the expression of the deacetylase SIRT1. As a crucial aging regulator, SIRT1 is thought to activate the downstream PI3K/AkT pathway. The activation of this pathway not only promotes the proliferation of ovarian granulosa cells and inhibits their apoptosis but also directly participates in regulating ovarian glucose metabolism homeostasis, providing the energy foundation for follicular development. The activation of these pathways has been observed to correlate with normalized secretion of gonadotropin-releasing hormone (GnRH) and luteinizing hormone (LH), contributing to the restoration of regular ovulatory cycles and normal sex hormone levels in these models [25,41].

### 2.2. Effects on the Male Reproductive System

#### 2.2.1. Testicular Structure and Spermatogenic Function

Fisetin has been reported to exert significant protective effects on testicular structure and spermatogenic function, particularly prominent in models of oxidative stress and toxic injury. Studies in rats have shown that Fisetin may alleviate monosodium glutamate (MSG)-induced testicular oxidative damage via central metabolic regulators, repair the structure of seminiferous tubules, increase sperm count, and reduce sperm morphological abnormalities [41]. In a rat model of arsenic-induced testicular toxicity, Fisetin significantly improved testicular histological structure by enhancing the activities of antioxidant enzymes (e.g., SOD, CAT, GPx) and reducing levels of the lipid peroxidation product TBARS and ROS. These improvements included increased seminiferous tubule diameter, epithelial height, and the number of various spermatogenic cells [17]. Furthermore, Fisetin has demonstrated protective effects in a rat model of testicular ischemia–reperfusion injury, where it appeared to alleviate spermatogenic epithelial disorganization, reduces the formation of multinucleated giant cells, and improves spermatogenesis-related indices [42].

The semen quality of breeding males is critical to the success of artificial insemination techniques. The protective effects of Fisetin on the seminiferous epithelium observed in rodent models suggest its potential to mitigate similar damage in breeding males. This could be particularly relevant during high-temperature seasons, where sensitive breeds such as sheep are prone to spermatogenic impairment due to heat stress. Although some wool-less sheep breeds exhibit a degree of heat tolerance, studies indicate that nutritional interventions are still necessary to maintain reproductive performance [27]. However, these applications require direct investigation in livestock species to determine effective doses and confirm efficacy. Translating effective dosages from rodent models to large animals requires careful consideration of allometric scaling based on factors such as metabolic body weight (e.g., mg/kg^0.75) and species-specific differences in drug metabolism. Current rodent studies have established effective dose ranges (e.g., 10–20 mg/kg/day), but establishing appropriate dose ranges for pigs and cattle will necessitate dedicated pharmacokinetic and pharmacodynamic studies in these target species to guide future clinical trials.

#### 2.2.2. Epididymis and Sperm Maturation

The epididymis is a crucial site where sperm acquire motility, complete functional maturation, and are stored. Fisetin has been reported to play a protective role in the maturation process of sperm within the epididymis and the storage environment, primarily reflected in improving epididymal sperm parameters, maintaining sperm DNA integrity, and regulating the local epididymal microenvironment.

In various injury models, Fisetin has been shown to significantly reverse the decline in sperm quality in the cauda epididymis caused by toxic substances or stress factors. Studies have shown that in a rat model of MSG-induced testicular toxicity, intraperitoneal administration of Fisetin (20 mg/kg) for 30 days significantly increased epididymal sperm count and reduced the rate of sperm morphological abnormalities [41]. This indicates that Fisetin not only protects testicular spermatogenic function but also safeguards the baseline quality of sperm entering the epididymis. In a mouse model of long-term scrotal heat stress, oral administration of Fisetin (10 mg/kg/day), whether during (preventive) or after (therapeutic) the heat stress period, effectively restored parameters such as sperm motility reduced by heat stress, with preventive administration showing better efficacy. The mechanism is associated with the downregulation of heat shock protein 72 (HSP-72) and NF-κB expression in the epididymis and testis, as well as the inhibition of oxidative stress and apoptosis [43]. Sperm DNA fragmentation is also a critical factor affecting male fertility and embryonic developmental potential. Scrotal heat stress significantly increases the sperm DNA fragmentation index in mice. Oral Fisetin treatment, particularly preventive administration, effectively reduces the elevation of the DNA fragmentation index caused by heat stress. This protective effect may be crucial for maintaining the integrity of the sperm’s genetic material and ensuring normal embryonic development post-fertilization [43]. The protective effect of Fisetin on epididymal function may involve its regulation of the secretory function of the epididymal epithelium and its redox balance. Its potent antioxidant activity may help scavenge excess ROS within the epididymal lumen, providing a stable, low oxidative stress environment for sperm maturation. Furthermore, by engaging energy-sensing and stress-resistance pathways, Fisetin may maintain the function of epididymal epithelial cells at the levels of cellular energy metabolism and stress resistance, indirectly safeguarding the microenvironment necessary for sperm maturation [41].

The maturation process of sperm in the epididymis is highly susceptible to oxidative stress, which can compromise the efficacy of cryopreservation [44]. The protective effects of Fisetin on epididymal sperm parameters and DNA integrity position it as an ideal candidate as an additive in semen extenders. In cryopreservation experiments involving boar and bull semen, the addition of Fisetin as an in vitro supplement to cryopreservation media is expected to reduce oxidative damage during the freeze–thaw process, enhance post-thaw sperm motility and fertilizing capacity, thereby providing higher-quality semen for artificial insemination [45]. It is critical to distinguish between this in vitro application and in vivo dietary supplementation. While the former involves direct addition to cryopreservation media, the latter would require oral administration to the breeding male, with the goal of systemically improving testicular health and baseline epididymal sperm quality. Additionally, Fisetin has been shown to inhibit the virulence of pathogens such as Actinobacillus pleuropneumoniae (APP), a causative agent of porcine contagious pleuropneumonia, suggesting its dual benefits in preventing epididymal infections and safeguarding the sperm maturation environment [46].

#### 2.2.3. Gonadal Hormone Levels

Normal gonadal hormone levels, particularly testosterone, are crucial for initiating and maintaining spermatogenesis and male secondary sexual characteristics. Fisetin can positively regulate the function of the HPG axis and promote sex hormone synthesis. It can significantly increase serum levels of GnRH, LH, follicle-stimulating hormone (FSH), and testosterone [41]. Additionally, it may promote testosterone synthesis by upregulating the expression of key steroidogenic enzymes such as steroidogenic acute regulatory protein (StAR), cholesterol side-chain cleavage enzyme (CYP11A1), steroid 17-alpha-hydroxylase (CYP17A1),3β-hydroxysteroid dehydrogenase (3β-HSD), and 17β-hydroxysteroid dehydrogenase (17β-HSD) [17]. Furthermore, Fisetin may support Leydig cell function via metabolic and stress-resistance pathways, further supporting testosterone synthesis and spermatogenesis [41,42].

Sex hormone levels directly regulate estrus expression and breeding success rates in livestock. The positive regulatory effects of Fisetin on the HPG axis observed in male models suggest its potential to influence the reproductive hormone status in females. This opens up a potential study direction for investigating whether Fisetin could aid in managing conditions like postpartum anestrus or seasonal anestrus in livestock. Future study is needed to evaluate the regulatory effects of Fisetin on estrous cycles and ovulation rates in female animal models and production settings.

## 3. Analysis of the Core Molecular Mechanisms Underlying Fisetin’s Reproductive Protective Effects

### 3.1. Antioxidant Stress and Anti-Inflammatory Pathways

The protective effects of Fisetin documented in the preceding sections converge on a limited set of core molecular pathways. Importantly, these pathways do not operate in isolation; rather, they form an integrated signaling network wherein the activation of one module (e.g., AMPK/SIRT1) reinforces others (e.g., Nrf2-mediated antioxidant defense) while suppressing detrimental cascades (e.g., NF-κB-driven inflammation). This section synthesizes these interconnected mechanisms into a cohesive framework, emphasizing their functional synergy rather than treating them as independent entities. In the reproductive system, oxidative stress is known to lead to damage in oocytes, sperm, and reproductive organs, thereby affecting fertility. As a potent flavonoid antioxidant, Fisetin’s reproductive protective effects are closely associated with the activation of the Nrf2 pathway. By activating the Nrf2/ARE signaling pathway, it has been reported to enhance the expression of antioxidant enzymes such as superoxide dismutase (SOD), catalase (CAT), and glutathione peroxidase (GPx), scavenges ROS in the body, reduce oxidative damage [47,48,49], and maintain redox homeostasis in reproductive cells.

NF-κB is a core transcription factor regulating the expression of inflammatory factors. In ovarian aging and PCOS, the NF-κB pathway is aberrantly activated, and Fisetin has been shown to directly inhibit this activation. In follicular stress models, Fisetin treatment significantly inhibited the NF-κB signaling pathway and the expression of its downstream target, cyclooxygenase-2 (COX-2), while also reducing levels of key pro-inflammatory cytokines such as TNF-α and IL-6 [50]. In ovarian ischemia–reperfusion injury, Fisetin inhibits NF-κB activation via the TLR4/MyD88 pathway, a key anti-inflammatory mechanism [26]. This anti-inflammatory action may be crucial for improving the endometrial immune microenvironment for embryo implantation and alleviating tissue inflammatory damage in the ovaries and testes. Despite the wealth of data supporting Nrf2 and NF-κB as key mediators of Fisetin’s effects, critical limitations must be acknowledged. Most studies rely on pharmacological inhibition or gene knockdown approaches in non-reproductive cell lines, with limited validation in primary reproductive tissues. Moreover, the relative contribution of Nrf2 activation versus direct ROS scavenging by Fisetin remains untested in most reproductive models. Importantly, several studies report Fisetin’s anti-inflammatory effects even under conditions where Nrf2 is not upregulated [26,50], suggesting the existence of Nrf2-independent pathways. The field would benefit from comparisons using genetic models to dissect causality. Additionally, most evidence comes from single-time-point measurements, leaving the temporal dynamics of pathway activation unknown.

### 3.2. Regulation of Autophagy and Apoptosis

The protective effects of Fisetin in the reproductive system are not only reflected in its antioxidant and anti-inflammatory responses but also in maintaining the health of germ cells and tissues by regulating autophagy and apoptosis. Autophagy clears damaged organelles and proteins, while apoptosis regulates programmed cell death; both are essential for reproductive tissue homeostasis [51,52]. Fisetin has been reported to promote autophagy by modulating key pathways such as PI3K/Akt, AMPK, and mTOR, and maintains cell survival and function by inhibiting excessive apoptosis [53,54,55].

Fisetin also alleviates endoplasmic reticulum (ER) stress during early embryogenesis. In porcine embryo culture, 0.1 µM Fisetin improved blastocyst formation and downregulated the ER stress marker GRP78, along with reducing autophagy- and apoptosis-related genes [56]. This suggests that Fisetin may create a more favorable developmental environment for early embryos by alleviating ER stress.

### 3.3. Regulation of Cellular Energy Metabolism and Hormonal Homeostasis

The reproductive protective effects of Fisetin also rely on its core regulation of cellular energy metabolism and hormonal homeostasis. The development and maturation of germ cells, as well as early embryo implantation, are highly energy-consuming processes, and their dysfunction is often directly linked to energy metabolism imbalance and subsequent hormonal disorders. Fisetin, by precisely regulating key energy sensors and metabolic hubs such as AMPK, SIRT1, and mammalian target of rapamycin (mTOR), may fundamentally improve the energy supply of reproductive cells, and it corrects hormonal imbalances under pathological conditions. This underlies its protective effects in scenarios such as PCOS, ovarian aging, and abnormal spermatogenesis.

In PCOS models, Fisetin has been reported to ameliorate insulin resistance and reduce abnormally elevated serum levels of testosterone and pro-inflammatory factors by activating the AMPK/SIRT1 pathway. This effect is thought to be related to the multidimensional synergistic action of activating the AMPK/PI3K/AkT-Nrf2 antioxidant axis and inhibiting the NLR family pyrin domain containing 3 (NLRP3)/NF-κB inflammatory pathway [50]. In aged oocytes, Fisetin significantly delays the decline in quality post-ovulation and enhances subsequent embryonic development potential by upregulating SIRT1 expression, enhancing mitochondrial function (e.g., Tfam, Co2, and ATP synthase protein 8 (Atp8)), and reducing ROS accumulation [14].

In terms of male reproduction, Fisetin’s ameliorative effects in oligoasthenospermia models involve the rebalancing of the liver kinase B1 (LKB1)/AMPK/mTOR/p70 Ribosomal Protein S6 Kinase (p70S6K) signaling pathway in testicular tissue. By inhibiting the overactive AMPK “energy-saving” pathway and promoting the mTOR “synthetic” pathway, it may help provide a suitable metabolic microenvironment for spermatogenesis while restoring physiological levels of FSH, LH, and T [16].

Furthermore, during early embryonic development, Fisetin may create more favorable metabolic conditions for blastocyst formation by alleviating ERS (e.g., downregulating GRP78 protein), enhancing mitochondrial function, and reducing oxidative damage [55].

In summary, the therapeutic potential of Fisetin in the reproductive system is best understood not as the sum of its effects on individual pathways, but as the outcome of its capacity to act as a central coordinator within an integrated signaling network. By simultaneously engaging antioxidant defense, inflammation control, energy sensing, and cell survival pathways—and facilitating cross-talk among them—Fisetin restores homeostasis under diverse pathological conditions.

Importantly, the above signaling pathways—including the energy-sensing AMPK/SIRT1 axis, the pro-survival PI3K/AkT cascade, the antioxidant Nrf2 pathway, and the inflammatory NF-κB pathway—do not function independently but rather constitute an integrated regulatory network. Fisetin acts as a central node within this network, orchestrating a coordinated cellular response. For instance, activation of AMPK/SIRT1 by Fisetin not only restores metabolic balance but also promotes Nrf2-mediated antioxidant defense while suppressing NF-κB-driven inflammation. Concurrently, modulation of the PI3K/AkT/mTOR axis ensures a balance between autophagy and apoptosis. This multi-pathway synergy enables Fisetin to simultaneously alleviate oxidative stress, inflammatory damage, metabolic dysregulation, and cell death, thereby providing robust and multifaceted protection to reproductive tissues and gametes.

**Figure 1:**
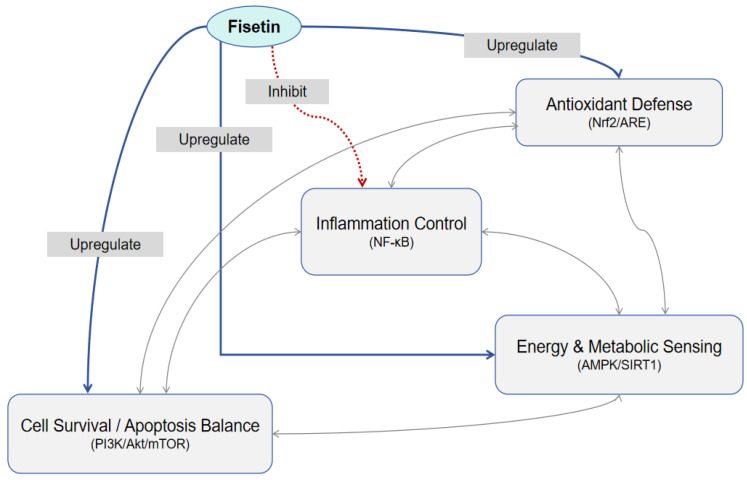
Schematic illustration of the core molecular mechanisms underlying Fisetin’s protective effects in the mammalian reproductive system. Fisetin exerts its beneficial actions by modulating a complex signaling network that includes (1) activating the Nrf2/ARE pathway to enhance antioxidant enzyme expression and scavenge reactive oxygen species (ROS); (2) inhibiting the TLR4/MyD88/NF-κB pathway to suppress the production of pro-inflammatory cytokines (e.g., TNF-α, IL-1β, IL-6); (3) regulating the AMPK/SIRT1/PI3K/Akt/mTOR axis to improve cellular energy metabolism, promote autophagy, and inhibit excessive apoptosis; and (4) restoring endocrine balance by positively regulating the hypothalamic–pituitary–gonadal (HPG) axis. Collectively, these coordinated actions protect ovarian follicles, oocytes, testicular seminiferous tubules, and epididymal sperm from various stressors, ultimately safeguarding fertility in both females and males. Abbreviations: ROS, reactive oxygen species; ARE, antioxidant response element; TLR4, toll-like receptor 4; MyD88, myeloid differentiation primary response 88; NF-κB, nuclear factor kappa-B; TNF-α, tumor necrosis factor-alpha; IL, interleukin; AMPK, AMP-activated protein kinase; SIRT1, sirtuin 1; PI3K, phosphoinositide 3-kinase; Akt, protein kinase B; mTOR, mammalian target of rapamycin; HPG, hypothalamic–pituitary–gonadal.

The integrated protective actions of Fisetin are summarized schematically in Figure 1, and key preclinical findings across different reproductive models are tabulated in Table 1.

## 4. From Basics to Clinical: Application Potential, Challenges, and Future Directions

### 4.1. Core Challenges in Clinical Translation

Although Fisetin has demonstrated multi-target protective potential for the reproductive system in preclinical studies [57], the primary obstacle on its path from laboratory to clinical application is its extremely low oral bioavailability. As a hydrophobic compound (Log P approximately 3.2), Fisetin has very poor water solubility (approximately 10.45 μg/mL) and is rapidly metabolized in the gastrointestinal tract, resulting in severely insufficient effective absorption and entry into systemic circulation. Its oral bioavailability is only approximately 44.1% [58,59]. This fundamental limitation restricts its efficacy when administered as a conventional oral formulation.

To overcome this bottleneck, nanotechnology-based drug delivery systems have become a research hotspot and the most promising solution [60]. The core of these strategies lies in improving Fisetin’s solubility, stability, and targeting capability through physical encapsulation or chemical modification. For ruminant applications, these nanocarriers offer an additional critical advantage: they may potentially protect Fisetin from premature degradation by the complex rumen microbiome. The encapsulation of Fisetin within liposomes, polymeric micelles, or solid lipid nanoparticles could act as a physical barrier, shielding the compound from microbial fermentation and enzymatic breakdown in the rumen, thereby enhancing its stability and enabling a greater proportion of the ingested dose to reach the small intestine for absorption.

Polymeric micelles, as an important class of carriers in nano-delivery systems, have shown significant advantages in enhancing the anti-ovarian cancer efficacy of Fisetin in vitro and in vivo [59]. Polymeric micelles made from monomethoxy polyethylene glycol-polycaprolactone (MPEG-PCL), when loaded with Fisetin, reduced its half-maximal inhibitory concentration (IC50) against SKOV3 ovarian cancer cells in vitro to 13.79 μg/mL, significantly enhancing the anti-tumor effect [61]. While these findings are from a cancer model, they serve as a proof-of-concept for the potential of nano-delivery systems to improve Fisetin’s bioavailability and efficacy, a principle that could be applied to non-pathological reproductive contexts. More importantly, intraperitoneal injection of Fisetin-loaded micelles at 50 mg/kg achieved a tumor growth inhibition rate of up to 70.7%, significantly superior to the free drug [59,61]. This enhanced efficacy stems from the micellar nanostructure fundamentally improving Fisetin’s hydrophobicity, thereby increasing the drug’s solubility and delivery efficiency to the tumor site.

Lipid-based nanosystems represent another major class of carriers, primarily including liposomes and solid lipid nanoparticles. Liposomes are composed of natural or synthetic lipids, forming one or more lipid bilayers. Utilizing their phospholipid bilayer structure, they can encapsulate both hydrophilic and hydrophobic drugs, protect the encapsulated molecules, and provide controlled drug release [62], significantly improving Fisetin’s solubility and promoting its cellular uptake [61]. Solid lipid nanoparticles, with a core of solid physiologically compatible lipids, offer controlled release and protection for unstable active molecules [63,64]. They not only possess higher physical stability but may also partially bypass hepatic metabolism through lymphatic absorption pathways. Kulbacka et al. [65] utilized a combination therapy involving Fisetin, photodynamic therapy, electroporation, and solid lipid nanoparticles to effectively deliver photosensitizers and anticancer drugs. This provides a promising avenue for improving the oral bioavailability of Fisetin.

Furthermore, inorganic nanoparticles, such as mesoporous silica nanoparticles, leverage their extremely high specific surface area and tunable pore structure to achieve high Fisetin loading [66]. The surface of such carriers is easily amenable to chemical modification, allowing conjugation with targeting molecules like folic acid or peptides. This enables active targeted delivery to specific cells (e.g., tumor cells) [61], potentially enhancing efficacy while reducing systemic toxicity to normal tissues.

Beyond delivery issues, the clinical translation of Fisetin faces other multidimensional challenges, including species differences and difficulties in dose extrapolation. Existing efficacy evidence primarily derives from animal models such as mice and rats. However, humans differ fundamentally from these animals in terms of the reproductive endocrine axis and drug metabolism pathways [67]. These differences make it difficult to directly apply doses determined in animal studies to humans. For example, the effective dose (1.25–2.5 mg/kg/day) [25] in a rat model of Polycystic Ovary Syndrome requires rigorous pharmacokinetic studies to confirm its human equivalent dose. Secondly, there is an extreme lack of long-term safety and reproductive-specific toxicological data. As a therapeutic agent intended for potential high-dose, long-term use, Fisetin’s clinical trials are still in the phase of evaluating long-term safety. Its potential effects on germ cell development and early embryonic development require systematic assessment. Finally, its mechanism of action is complex, and the lack of specific pharmacodynamic biomarkers capable of dynamically reflecting its integrated biological activity within the reproductive system poses challenges for the precise evaluation of clinical efficacy.

### 4.2. Future Perspectives in Clinical Research

To advance Fisetin, a natural flavonoid compound with broad pharmacological activities, from a promising molecule to a clinically viable therapeutic tool, future research must formulate a coherent and multi-layered strategy.

The core starting point of this strategy is to fundamentally overcome its inherent physicochemical limitations—namely, the development of intelligent novel delivery systems. Its poor water solubility and oral bioavailability are the primary obstacles hindering clinical translation [68]. Next-generation delivery technologies should transcend simple solubilization and focus on designing nanocarriers capable of specifically responding to the local microenvironment of reproductive organs (e.g., specific pH, enzyme activity, or redox state). This would enable precise drug release at lesion sites such as the ovaries or testes, thereby enhancing efficacy while minimizing systemic side effects. A study on neurogenic erectile dysfunction indicated that Fisetin exerts protective effects by upregulating the Peroxisome proliferator-activated receptor-γ (PPAR-γ) pathway, providing a clear molecular target for designing delivery systems tailored to the local microenvironment of the testis or penile corpus cavernosum [69].

Having addressed the delivery bottleneck, research focus should shift towards rigorously designed early-phase clinical studies. There is an urgent need to initiate Phase clinical trials targeting reproductive system diseases. The primary objectives are to elucidate the pharmacokinetic characteristics and safety profile of Fisetin in humans using different advanced delivery systems [68]. Trials could prioritize areas with clearly unmet clinical needs for proof-of-concept studies. For example, based on its mechanisms involving pathways like AMPK/SIRT1 and NF-κB, Fisetin could be explored as an adjunctive treatment for improving insulin resistance, hormonal disturbances, and ovarian inflammation in patients with PCOS, or as a potential intervention for male oligoasthenospermia. Simultaneously, further evidence related to these pathways could be collected within the clinical trials.

Given the pathological complexity of reproductive system diseases, in-depth exploration of combination strategies involving Fisetin and other established therapies holds significant promise. For instance, combining Fisetin with the insulin sensitizer metformin for PCOS might synergistically improve glucose metabolism while adding Fisetin’s unique antioxidant, anti-inflammatory, and direct ovarian protective effects [50,70]. Alternatively, its integration with assisted reproductive technologies could be considered, exploring the feasibility of adding safe concentrations of Fisetin to embryo culture media to combat oxidative stress and improve oocyte quality and embryonic developmental potential. The concept of combination therapy has already gleaned insights from other disease models; for example, the combination of Fisetin with hydroxychloroquine has shown synergistic potential in improving metabolic parameters [71].

Ultimately, to achieve true individualized precision medicine, future research must focus on establishing a dynamic and precise panel of pharmacodynamic biomarkers. This involves discovering and validating biomarkers capable of reflecting Fisetin’s action on reproductive target organs in real-time. These biomarkers could be derived from its core pathways of action, such as antioxidant indicators (e.g., downstream products of Nrf2), specific inflammatory factors (e.g., IL-1β, IL-6), key reproductive hormone levels (e.g., FSH, LH, T), or direct parameters of germ cell quality. This biomarker panel should be integrated throughout the entire process, from basic research to clinical validation, serving to guide patient stratification, optimize dosing regimens, and enable real-time efficacy monitoring. This would ultimately facilitate the evidence-based integration of Fisetin into the clinical practice of reproductive health management.

### 4.3. Future Perspectives in the Field of Animal Reproduction

The successful application of Fisetin in medical models has opened new research directions for its use in animal reproduction. Given the urgent demands of modern livestock farming for reproductive efficiency and genetic resource conservation, future targeted research could focus on the following areas for exploration.

In the semen cryopreservation of economically important animals such as pigs, cattle, and sheep, oxidative stress is a primary cause of decreased sperm motility and DNA damage. Leveraging its potent reactive oxygen species scavenging ability and activation of the Nrf2 pathway, Fisetin could be investigated as a novel natural cryoprotectant additive to semen extenders [17,47]. Systematic research is needed to determine its optimal concentration, exposure time, and synergistic effects with existing cryoprotectants, with its actual efficacy to be validated through in vitro fertilization and artificial insemination trials. For oral administration in ruminants, future research must also evaluate the protective efficacy of nano-encapsulated Fisetin against rumen degradation. Comparative studies investigating the pharmacokinetic profiles and ultimate bioavailability of free versus nano-encapsulated Fisetin in cattle and sheep will be essential to validate the practical utility of these advanced delivery systems in livestock production.

Drawing on the mechanisms by which Fisetin delays oocyte aging and improves mitochondrial function, its potential effects on cumulus expansion, first polar body extrusion rate, and subsequent embryonic developmental potential can be evaluated in in vitro maturation (IVM) systems [22,72]. Additionally, exploring the safe window for its supplementation in in vitro culture (IVC) systems could enhance blastocyst formation rates and embryo quality.

For metabolic stress and reproductive disorders faced by high-yielding dairy cows and multiparous sows [27,28], Fisetin could be developed as a functional feed additive for long-term feeding trials to evaluate its effects on reproductive performance. Its effects on estrus synchronization rates, ovulation number, conception rates, and embryo survival should be monitored. Combined with assessments of serum hormone levels, ovarian antioxidant indicators, and uterine environmental parameters, its application value under actual production conditions can be evaluated.

Existing dosage data are primarily derived from rodent studies; therefore, future pharmacokinetic and toxicological studies in large animals such as pigs and cattle are necessary to determine its effective dose range and safety window. Concurrently, attention should be paid to the residue and metabolic patterns of Fisetin in animals to provide a scientific basis for its standardized application in livestock production, contributing to green, safe, and precision livestock farming.

## 5. Conclusions

Current evidence suggests that Fisetin, as a multi-target, multi-pathway natural active molecule, has demonstrated broad protective potential in the mammalian reproductive system. The present review extends beyond a descriptive summary of this evidence by critically evaluating research gaps and by delineating a translational framework that connects mechanistic insights from rodent models to potential applications in both human reproductive medicine and livestock fertility management. Findings from preclinical studies indicate that, through core mechanisms such as antioxidant, anti-inflammatory effects, regulation of energy metabolism, and hormonal homeostasis, it may improve ovarian function, oocyte quality, testicular spermatogenesis, and sperm parameters, showing interventive effects in various reproductive endocrine disease models. These characteristics suggest inherent potential advantages of being natural and safe compared to traditional hormone therapies.

However, significant bottlenecks remain in the translation of Fisetin from basic research to clinical practice. Its extremely low oral bioavailability is the primary limitation, with nanotechnology-based drug delivery systems regarded as a key strategy to overcome this hurdle. Furthermore, current evidence is predominantly derived from animal models, necessitating future urgent clinical trials targeting human reproductive system diseases to validate its safety, effective dosage, and specific efficacy. Its complex mechanisms of action also require future research to deeply elucidate the synergistic relationships among different pathways and identify biomarkers capable of dynamically monitoring its reproductive protective effects.

The added value of this review lies in its critical and integrative approach, which not only synthesizes existing knowledge but also identifies critical bottlenecks and proposes a structured framework for future research. By emphasizing the translational pathway—from overcoming bioavailability hurdles to exploring applications in both human medicine and livestock production. This review provides a distinct roadmap that moves beyond a mere summary of existing literature. Looking ahead, through the future development of intelligent targeted delivery systems, the design of combination strategies with existing therapies, and rigorous clinical translational research, Fisetin may hold promise as an innovative natural intervention for challenges such as reproductive endocrine disorders, reproductive aging, and infertility. However, its application value needs to be fully realized through systematic follow-up investigations. For the field of animal reproduction, the path forward must extend beyond in vitro and rodent studies to include well-designed field trials in livestock. These trials are essential to validate the efficacy of Fisetin, particularly using advanced nano-formulations, under real-world production conditions. Key outcomes such as conception rates, litter sizes, and semen quality post-cryopreservation must be assessed to determine its economic viability and practical utility in large-scale animal production systems.

Furthermore, although research on Fisetin in the field of animal reproduction is still in its early stages, its potential in sperm protection, oocyte maturation, and regulation of female livestock reproduction has begun to emerge. Through interdisciplinary studies, Fisetin may be expected to transition from the laboratory to livestock production, offering a safe and natural solution for improving reproductive efficiency in domestic animals and ensuring the sustainable utilization of genetic resources.

## Figures and Tables

**Table 1:** Summary of key preclinical studies demonstrating the protective effects of Fisetin on mammalian reproductive function.

Model/Condition	Species/Cell Type	Fisetin Dose & Route	Key Reproductive Outcomes	Proposed Molecular Mechanism(s)	Ref.
Postovulatory Oocyte Aging	Mouse	10 μM (in vitro)/20 mg/kg (in vivo)	Delayed oocyte aging; improved mitochondrial function and embryonic development	↑ Sirt1, Tfam, Co2, Atp8; ↓ ROS, lipid peroxidation	[14]
Ovarian Aging	Laying Hen	100–400 mg/kg (diet)	Enhanced ovarian antioxidant capacity; promoted cell proliferation	↑ Antioxidant enzyme activity; ↓ MDA, ROS	[15]
Polycystic Ovary Syndrome (PCOS)	Rat (Letrozole-induced)	2.5 mg/kg/day (oral)	Improved insulin resistance; reduced androgens; restored ovarian morphology	↑ AMPK/SIRT1, PI3K/Akt-Nrf2; ↓ NLRP3/NF-κB	[50]
Ovarian Ischemia–Reperfusion Injury	Rat	20, 40 mg/kg (i.p.)	Reduced tissue damage; decreased inflammatory infiltration	↓ TLR4/MyD88/TRAF6, NF-κB, TNF-α, IL-1β, IL-6; ↑ SOD, GSH	[26]
Monosodium Glutamate (MSG) Toxicity	Rat	20 mg/kg/day (i.p., 30 d)	Improved seminiferous tubule structure; increased sperm count and motility	↑ SIRT1/AMPK, T, LH, FSH; ↓ oxidative stress	[41]
Arsenic-Induced Toxicity	Rat	20 mg/kg/day (oral gavage)	Restored testicular histology; enhanced spermatogenesis; increased testosterone	↑ StAR, CYP11A1, 3β-HSD; ↑ SOD, CAT, GPx; ↓ TBARS	[17]
Scrotal Heat Stress	Mouse	10 mg/kg/day (oral)	Restored sperm motility; reduced sperm DNA fragmentation	↓ HSP-72, NF-κB, oxidative stress, and apoptosis	[43]
Testicular Ischemia–Reperfusion	Rat	20 mg/kg (i.p.)	Alleviated epithelial disorganization; improved spermatogenesis	↑ SIRT1/AMPK; ↓ oxidative stress and apoptosis	[42]
Oxidative Stress	Porcine Embryo	0.1 μM (in vitro culture)	Improved blastocyst formation rate	↓ GRP78, ER stress; ↓ autophagy/apoptosis-related genes	[56]

Abbreviations: ↑, increase/upregulate/activate; ↓, decrease/downregulate/inhibit; i.p., intraperitoneal; d, day; ROS, reactive oxygen species; MDA, malondialdehyde; SOD, superoxide dismutase; T, testosterone; LH, luteinizing hormone; FSH, follicle-stimulating hormone; CAT, catalase; GPx, glutathione peroxidase; ER, endoplasmic reticulum.

## Data Availability

No new data were created or analyzed in this study. Data sharing is not applicable to this article.
